# Difficulties in assessing cytomegalovirus-associated gastric perforation in an HIV-infected patient

**DOI:** 10.1186/1471-2334-5-28

**Published:** 2005-04-13

**Authors:** Bruno Mégarbane, Dabor Résière, Jacqueline Ferrand, Laurent Raskine, Kouroche Vahedi, Frédéric J Baud

**Affiliations:** 1 Medical and Toxicological Intensive Care Unit, Lariboisière Hospital, Paris, France; 2 Department of pathology, Lariboisière Hospital, Paris, France; 3 Department of Bacteriology and Virology, Lariboisière Hospital, Paris, France; 4 Department of Gastro-enterology, Lariboisière Hospital, Paris, France

## Abstract

**Background:**

Active Cytomegalovirus (CMV) infection is a common complication in advanced symptomatic Human Immunodeficiency Virus (HIV) infection. CMV-induced intestinal perforations are hard to diagnose and may be observed throughout the gastrointestinal tract. Isolated stomach perforation is exceptional.

**Case presentation:**

A 47-year-old man was admitted to our intensive care unit with multiorgan failure. Gastrointestinal endoscopic examination showed erythematous gastritis but normal duodenum and colon. CMV blood culture was positive. Histologic examination of a gastric biopsy showed inflammatory infiltrate and immunostaining typical intranuclear CMV inclusion bodies. Concomitant abdominal CT scan disclosed large peripancreatic hypodensities without pneumoperitoneum. The patient died despite supportive therapies and ganciclovir infusion. Postmortem examination showed a 4-cm gastric perforation adhering to the transverse colon and liver, with a thick necrotic inflammatory coating around the pancreas. The whole GI tract, except the stomach, was normal. As other causes, especially *Helicobacter pylori *infection could be ruled out, a causal relationship between CMV and gastric disease was assumed.

**Conclusion:**

CMV may be responsible for gastric perforations, with difficulties in assessing the diagnosis. Early diagnosis based on cautious endoscopy and histopathologic examination is needed to make a favorable outcome possible.

## Background

Cytomegalovirus (CMV) is frequently isolated from immunosuppressed patients with HIV infection, organ or bone marrow transplants, malignancies or immunosuppressive medications. CMV may cause disseminated diseases and is generally predictive, in HIV-infected patients, of poor long-term survival reflecting severe immunosuppression [1]. In these patients, CMV gastrointestinal (GI) infection, including colitis, esophagitis, and gastritis [2,3]. Clinical signs are usually sparse, making the diagnosis difficult. Although CMV-induced intestinal perforation has been observed in the patients with advanced immunodeficiency, isolated gastric perforation remains a rare event. We report here the fatal case of an HIV-infected patient, who presented a CMV-associated gastric perforation.

## Case presentation

**Figure 1:**
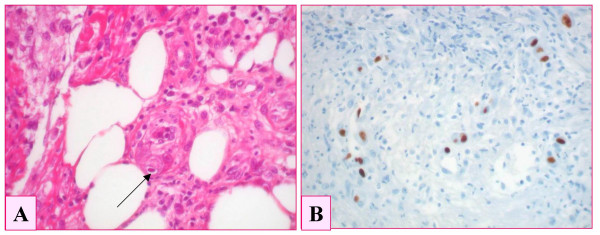
Biopsy specimen of gastric glandular epithelial mucosa showing the presence of intranuclear cytomegalic inclusion bodies (1A, arrow), together with edema and inflammatory infiltrate. CMV infection was confirmed using immunohistochemistry, with a specific anti-CMV antibody (DAB-peroxydase) (1B).

**Figure 2:**
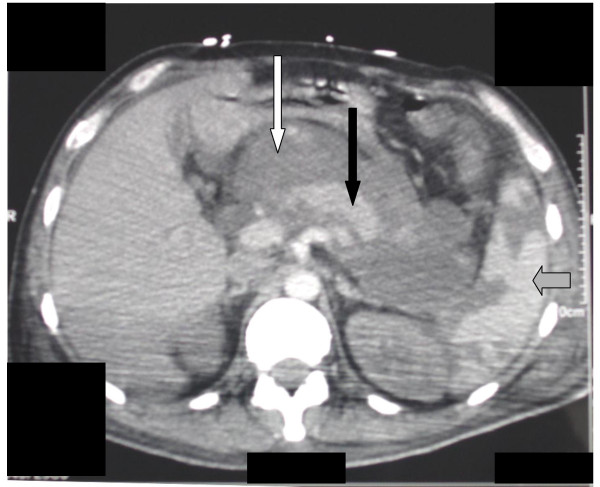
Abdominal CT scan showing the presence of large and heterogeneous hypodensities (open arrow), surrounding the pancreas (filled arrow) and mildly enhanced following contrast infusion. The spleen (gray arrow) was patchy, demonstrating ischemic zones.

A 47-year-old man was hospitalized in our intensive care unit (ICU) for generalized seizures and hypotension. Three days before admission, he suffered from fatigue, fever, vomiting, abdominal pain and lower GI hemorrhage. He was homeless and a chronic alcoholic. Physical examination showed general status impairment, with a temperature of 36.5°C and a Glasgow Coma Score of 10. No focal neurological deficit was noted. His blood pressure was 80/26 mmHg, his heart rate 106 /min and his respiratory rate 36 /min. His abdomen was tender with hepatomegaly. Usual blood tests showed a creatinine of 413 μmol/l (N <120), white blood cell count of 15.1 × 10^6 ^/l (N: 4 × 10^6^-10 × 10^6^) with 85% polymorphonuclear leukocytes, hemoglobin of 8.5 g/dl (N: 13–18) and platelets 178 × 10^6 ^/l (N: 150–400). He presented with severe lactic acidosis (arterial pH 7.21 (N: 7.34–7.45), PaCO_2 _3.7 kPa (N: 4.3–6.0), blood bicarbonate 11 mmol/l (N: 20–26), and plasma lactate 22 mmol/l (N: 1–2)). His prothrombin time was 9% (N>75%), fibrinogen 3.72 g/l (N: 2–4), factor V 25% (N >75%), and factors II + VII 5% (N >75%). Other tests showed an AST of 4,480 UI/l (N<50), an ALT of 2,340 UI/l (N<50), bilirubin 67 μmol/l (N<17), LDH 24,500 UI/l (N<275), lipase 11,820 UI/l (N<200), and creatine phosphokinase 3,330 UI/l (N<170). Abdominal ultrasonography showed an enlarged liver without ascites. The chest X-ray, electrocardiogram, cerebral CT scan were unremarkable. The patient was intubated. He received IV infusions of norepinephrine (6 mg/h), dobutamine (15 μg/kg/min), omeprazole (80 mg/day), N-acetylcysteine (300 mg/kg over 20 h), cefotaxime (3 g/day) and metronidazole (1.5 g/day). Continuous hemodiafiltration was started. Cultures of blood, urine, stools and tracheal aspiration were negative. Toxicological screening tests, including acetaminophen, ethanol, ethylene glycol, and methanol were negative. HIV-1 ELISA serology was positive, CD4+ lymphocytes count 160 × 10^6 ^/l (N >700), and HIV-1 RNA level was 5.8 × 10^5 ^copies/ml. Bronchoalveolar lavage (BAL) showed 2.5 × 10^8 ^cells/l with 58% lymphocytes. A diagnosis of *Mycobacterium tuberculosis *infection was rapidly assessed by an amplification assay (Amplified Mycobacterium Tuberculosis Direct Test, Gen Probe, California) and retrospectively confirmed by BAL cultures. Isoniazid, rifampicin, ethambutol, and pyrazinamide were started on day 3. Endoscopic examination showed grade II esophageal varices, moderate peptic esophagitis, erythematous gastritis with cardial superficial ulcerations without active bleeding, and normal duodenum and colon. CMV culture using MRC5 cells inoculation was positive in blood but not in BAL. CMV amplification assay was negative in the cerebrospinal fluid. Histopathologic analysis of a gastric biopsy showed edema, inflammatory infiltrate and typical intranuclear cytomegalic inclusion bodies in endothelial cells in mucosa. Immunohistochemistry with a specific anti-CMV antibody (E13, Argène, France) confirmed active CMV infection of the stomach (Fig. 1). Specific staining and culture were negative for *Helicobacter pylori*. As soon as these results were known (on day 10), ganciclovir was started. Concomitant abdominal CT scan disclosed large and heterogeneous peripancreatic hypodensities, mildly enhanced following contrast infusion (Fig. 2). However, their exact etiology remained undetermined. The patient developed hospital-acquired *Pseudomonas aeruginosa *pneumonia and his condition worsened. He died 30 days after admission. Postmortem examination showed a large, well-circumscribed gastric perforation with a diameter of 4 cm. The stomach was solidly adhered to the transverse colon and to the lower face of the liver. There was a thick necrotic and hemorrhagic inflammatory coating around the pancreas, which, in contrast, appeared macroscopically normal. The whole GI tract, except the stomach, was normal.

CMV infection of the GI tract can cause severe damage. Isolated involvement of the stomach is possible, although rare [3,4]. Symptoms are not specific, including unexplained fever, dysphagia, sharp or postural epigastric pain, vomiting, diarrhea, and GI bleeding. Characteristic upper endoscopic findings are edema, congestion, mucosal ulcerations, multiple punched-out gastric ulcers, erosions, and upper GI hemorrhage [5]. However, findings may be mild showing congestion and thickening or be atypical, with hemorrhaged necrotic gastritis or pseudo-tumors [6]. Multiple mucosal biopsies are needed to detect CMV [7]. In HIV-infected patients, CMV is significantly associated with chronic active gastritis or gastroduodenal ulcers [7-9], in contrast with healthy adults, in whom symptomatic CMV gastric infection is exceptional [10]. Thus, early recognition of CMV GI infection, including blood cultures and cautious GI endoscopic evaluation, may allow for adequate therapy, preventing complications, such as life-threatening bleeding or perforation [3,11].

CMV infection activates endothelial cells and leukocytes, altering GI microcirculation and inducing extensive vasculitis, thrombotic vascular occlusion, necrosis, and ischemic perforation [12]. The most common reported locations of intestinal perforation are the colon (53%) [13-15], the distal ileum (40%) [16,17], and the appendix (7%) [18]. CMV-associated perforation of the stomach is a rare presentation [19]. To our knowledge, isolated CMV-induced gastric perforation has only been reported in non-HIV organ transplant recipients [20]. In our patient, CMV-associated gastric perforation, as a presenting manifestation of HIV infection, was responsible for multiorgan failure. The diagnosis was difficult, in the absence of acute abdomen and pneumoperitonium. Consistently, initial endoscopic examination missed the gastric perforation, since the hole in the stomach was probably filled in by peritoneum. Moreover it is possible that a suboptimal endoscopic evaluation of the upper GI tract was due to the patient's initial unstable condition and the performance of the gastroscopy in such a critical situation. In contrast, concomitant abdominal CT scan showed large hypodense images surrounding the pancreas, attributed after postmortem examination, to the hemorrhagic and necrotic inflammatory materials that filled in the gastric perforation.

Alternative causes of gastric ulcer, including *Helicobacter pylori *infection, should be discussed. Indeed, CMV is an opportunistic virus and may thus appear in previously damaged tissues. However, a recent study suggested that CMV, rather than *Helicobacter pylori*, may be the main causative pathogen of peptic ulcers in HIV-infected patients, in comparison to non HIV-infected ones [9]. In our case, histopathologic findings and cultures were negative for *Helicobacter pylori*. Moreover, CMV inclusion bodies, which were observed on initial pathological findings, clearly indicated the existence of a CMV disease at the patient's admission.

Usually, resolution of symptoms and endoscopic findings is obtained with IV ganciclovir or foscarnet. Combined antiretroviral therapies may also be effective, even without specific treatment of CMV [21]. However, in the case of perforation, despite immediate surgical resection and antiviral therapy, mortality rate remains elevated (> 80%), due to elevated operative mortality and increased postoperative complications [17,18]. In our case, ganciclovir therapy successfully reduced CMV viral load before the occurrence of death, which resulted from misdiagnosed gastric perforation.

## Conclusion

This dramatic presentation demonstrates that perforation may complicate CMV gastric infection in HIV-infected patients. Physicians should be aware that early diagnosis, based on cautious GI endoscopy and histopathologic examination of GI biopsy specimen is needed to make a favorable outcome possible.

## Competing interests

The author(s) declare that they have no competing interest.

## Authors' contributions

BM, DR and FJB carried out the clinical study and drafted the manuscript. JF carried out the autopsy and the postmortem findings. LR carried out the microbiological studies. KV performed the gastrointestinal endoscopy. All the authors read and approved the final manuscript.

## Pre-publication history

The pre-publication history for this paper can be accessed here:


